# Anesthesia Practice: Review of Perioperative Management of H-Type Tracheoesophageal Fistula

**DOI:** 10.1155/2019/8621801

**Published:** 2019-11-03

**Authors:** Bret Edelman, Bright Jebaraj Selvaraj, Minal Joshi, Uday Patil, Joel Yarmush

**Affiliations:** ^1^Department of Anesthesiology, New York Presbyterian—Brooklyn Methodist Hospital, Brooklyn, NY 11215, USA; ^2^Department of Pediatrics (Division of Neonatal—Perinatal Medicine), Icahn School of Medicine at Mount Sinai, New York, NY 10029, USA

## Abstract

Tracheoesophageal fistula (TEF) is a rare congenital developmental anomaly, affecting 1 in 2500–3000 live births. The H-type TEF, consisting of a fistula between the trachea and a patent esophagus, is one of the rare anatomic subtypes, accounting for 4% of all TEFs. The presentation and perioperative management of neonates with H-type TEFs and all other TEFs are very similar to each other. Patients present with congenital heart disease and other defects and are prone to recurrent aspirations. A barium esophagogram or computed tomography of the chest is a common means to the diagnosis, and surgical repair is carried out through either a cervical approach or a right thoracotomy. During operation, anesthetic management is focused on preventing positive pressure ventilation through the fistula in an attempt to minimize gastric distension. For patients with H-type TEFs, because of the patent esophagus, symptoms are often less severe and nonspecific, resulting in subtle yet important differences in their diagnostic workup and management. This review will cover the finer details in the diagnosis and perioperative anesthetic management of TEF patients and clarify how H-type TEF distinguishes itself from the other anatomic subtypes.

## 1. Introduction

Tracheoesophageal fistula (TEF) is a congenital developmental anomaly that affects approximately 1 in 2500–3000 live births [1]. TEF is classified into five subtypes based on the location of fistula and the presence or absence of esophageal atresia. We will focus our review on isolated TEF without esophageal atresia (type H). Lamb reported the first case of H-type TEF in 1873. The first surgical repair was reported by Imperatori in 1939. The exact pathogenic mechanism of TEF is unclear. It is thought to be due to incomplete separation of the tracheal bud from the primitive foregut during the 4^th^ or 5^th^ week of embryogenesis. The incidence of associated congenital anomalies is lower in H-type TEF compared to patients with other types of TEF.

## 2. Presentation and Diagnosis

Symptoms are highly variable, and diagnosis is often difficult with single examination. Diagnosis may be delayed up to adolescence, may be even up to adulthood in some patients. Clinicians should have a high index of suspicion to establish the diagnosis of H-type TEF. In a review of 102 patients, diagnosis was delayed past the first month of life in 31% [2]. Diagnosis is often delayed as the esophagus remains intact without interruption. Tracheal opening mostly lies above the esophageal opening. This allows esophageal content to pass preferentially in to the stomach. Another theory for late diagnosis is that there is a mucosal flap which acts like a valve which closes during swallowing or the lumen of fistula which closes during swallowing due to spasm of the muscular layer [3, 4].

Antenatal ultrasound findings such as polyhydramnios and small/absent fetal stomach bubble are less reliable with a positive predictive value of only 44%. Incidence of prematurity is less in H-type TEF given their ability to swallow as a fetus is intact and polyhydramnios less likely [5]. Common clinical symptoms include cyanotic spells, choking episodes while feeding, recurrent pneumonia, and intermittent abdominal distension with excessive flatulence. Oftentimes, these symptoms are misdiagnosed as gastroesophageal reflux disease.

Investigations help to establish the diagnosis, plan surgical approach, and screen for associated congenital anomalies. Esophagography, although useful, may need repeated attempts to diagnose H-type TEF. Contrast studies like barium swallow and computed tomography (CT) may increase the risk of aspiration and lung injury. As the fistula is located higher, it may be missed at endoscopy or during an inexpertly performed contrast study. A retrospective analysis by Jiangtao Dai and colleagues looked at 31 patients diagnosed with H-type TEF in a period of 17 years. Out of 31 cases, 16 were diagnosed by iodine oil contrast examination of the esophagus, 11 were diagnosed by fiberoptic bronchoscopy (with methylthioninium chloride injection) combined with esophagography, and 4 were diagnosed by 3D CT reconstruction [6]. Bronchoscopy helps identifying the location, size, and number of fistula as well as other potential abnormalities like tracheomalacia or vascular rings. It can also be used to cannulate the fistula with a vascular guidewire or Fogarty or ureteral catheter, which can facilitate intraoperative identification [7].

## 3. Treatment Options and Approach

Endoscopic procedures like fibrin occlusion, sclerotherapy, electrocautery, or laser coagulation have been described for the purpose of ligating TEF. These procedures, although demonstrated lower morbidity and mortality, have high recurrence rates [8]. Surgery has therefore remained the standard treatment for TEF.

Open surgical repair is performed by a cervical approach or transthoracic approach (right thoracotomy). A cervical approach is recommended for fistulas located above the level of T2, which represent 70% of all H-type TEFs [8–11].

In recent years, thoracoscopic approach has been attempted successfully on tracheal pathology, including H-type TEFs, situated between the thoracic inlet and the carina [12]. Advantages of the thoracoscopic approach include great surgical exposure and decreased morbidity. The lung experiences less surgical trauma and postoperative chest tube can be avoided, permitting faster extubation and recovery.

## 4. Preoperative Anesthetic Management

A complete physical examination is essential during preoperative assessment to look for associated congenital anomalies like cardiac defects, imperforate anus, and genital malformations [13]. 12-lead electrocardiogram and echocardiogram should be done to diagnose/manage associated congenital heart disease and also to help optimize congestive heart failure. The nature of a cardiac lesion can also dictate management. Neonates with nonductal-dependent cardiac lesions can proceed to the operating room for a primary repair. For neonates with ductal-dependent lesions, there are two options: delaying the repair and beginning with a cardiac palliation procedure or proceeding with the repair while maintaining a prostaglandin (PGE_1_) infusion [14].

Lumbar ultrasound should be done in all patients with the sacral dimple. Acid suppression medications and antibiotics should be started at least 24 hours preoperatively if aspiration is suspected [15]. Patients may have poor lung compliance due to repeated attacks of pneumonia due to aspiration. Care should be taken to optimize respiratory status as much as possible before surgery.

Aside from managing lung compliance and concurrent congenital cardiac anomalies, preoperative care consists of routine preoperative blood tests, humidified oxygen, heat, glucose-containing maintenance fluids, maintenance of temperature, and correction of any electrolyte or coagulation abnormalities. With regard to blood tests, a type and cross should be obtained, even though the need for transfusions with surgical repair is uncommon. Finally, blood for genetic studies should also be collected so as to provide information about long-term prognosis to parents as quickly as possible [16].

## 5. Intraoperative Anesthetic Management

In addition to standard ASA monitors, intra-arterial line is recommended for intraoperative monitoring [17, 18]. Airway management depends on the location and size of the fistula. Traditional teaching is to avoid positive pressure ventilation including mask ventilation till the fistula is ligated to prevent gastric distension, which can restrict ventilation and increase the risk of gastric perforation. But recent experience shows that controlled ventilation is relatively safe in patients with small fistula without respiratory compromise [19]. One review of 61 cases at Children's Hospital in Oakland found that fistulas ≤3 mm in diameter presented no problems for positive pressure ventilation [20].

Positioning is dependent on the surgical approach. For a thoracoscopy, patients are placed in a 3/4 prone position, with the right side elevated to 45° and the right arm placed over the head [21]. Given the possibility of easy displacement of the endotracheal tube in the short airway of the neonate, bronchoscopic confirmation of proper tube placement should be attained after the patient is positioned prone [22]. For a right thoracotomy, positioning is left lateral decubitus. For the cervical approach, the patient lies supine with the head slightly extended.

Positioning of the endotracheal tube with its end lying distal to the fistula is essential to avoid gastric distension. Traditionally, the tube has been placed past the carina, into the right main bronchus. Once unilateral breath sounds are confirmed, the tube is pulled back until breath sounds become bilateral. The tube should be oriented with the Murphy eye/bevel situated anteriorly, this will permit the shaft of the tube to occlude the fistula, which is located on the posterior tracheal wall [17]. Proper positioning of the tracheal tube may be difficult to both achieve and maintain if the fistula is situated near the carina. Precise placement may be enhanced using a fiberoptic bronchoscope for guidance. If a fiberoptic bronchoscope is unavailable, and the neonate has a gastrostomy tube, placement may be confirmed by placing the end of the gastrostomy tube under a water seal. The presence of bubbling with ventilation indicates the passage of gas through the fistula, which in turn identifies positioning of the tip of the tube proximal to the fistula. In this scenario, the endotracheal tube should be withdrawn after its endobronchial placement until bubbling occurs and then readvanced until the bubbling subsides [20]. A capnograph inserted into the gastrostomy tube can produce the same result by monitoring end tidal carbon dioxide waveforms [23].

For very large TEFs, prevention of ventilation through the fistula may require its occlusion with either a cuffed endotracheal tube or a 2 or 3 Fr Fogarty catheter placed before the tracheal tube under bronchoscopic visualization. With the patient supine, the catheters are more likely to pass through the fistula, located posteriorly and in a dependent position. Following placement, the balloon is inflated, the bronchoscope is removed, and the placement of the endotracheal tube is carried out in a standard fashion [24]. Advancement of the Fogarty catheter through the fistula can also be carried out through a gastrostomy if one has been placed. The use of Fogarty catheters, however, can be problematic, in addition to being technically complex. Neonates with respiratory compromise may be unable to tolerate the interruptions in ventilation that occur if a rigid bronchoscope is used to pass the catheter. The catheters can be difficult to place and can dislodge, causing central airway obstruction [25, 26]. Catheters can also damage the esophageal mucosa at the site of balloon inflation [26].

Another option is one-lung ventilation in the left or right main-stem bronchus until the fistula is ligated. It can be difficult however to find a tracheal tube that adequately fits within the trachea without being too large for the main-stem bronchus and placing the patient at risk for bronchial edema due to the overly tight fit. One-lung ventilation can also be achieved by the Fogarty catheter placed in the right main bronchus in addition to the one placed in the fistula. Both maneuvers should be performed under bronchoscopic guidance [17, 24, 27, 28]. Surgical insufflation of CO_2_ (5-6 mmHg) into the right hemithorax can be expected to create hypercapnia, acidosis, and hypoxia as the right lung collapses [28]. In one case series of 8 TEF repairs done thoracoscopically, all but one required 100% oxygen to maintain SpO_2_ >85% when the right lung was collapsed during one-lung ventilation [22]. During the period of lung collapse, the anesthesiologist should be mindful of a sudden increase in ETCO_2_, which can be a sign of possible surgical trauma to the collapsed lung and entrainment of insufflating gas [25].

Surgeons may request to apply traction on a Fogarty catheter or guidewire placed in the fistula to help identify its location intraoperatively [10]. In one case, traction on a guidewire placed through the H-type TEF with both ends brought out through the mouth lifted a low cervical/high thoracic fistula enough cephalad to enable the surgeon to repair it with a less morbid cervical approach instead of a thoracotomy [29]. Surgeons may also request reduction of delivered tidal volumes to minimize mediastinal movement and optimize conditions for esophageal repair.

Positive pressure ventilation may be initiated once the fistula has been surgically ligated or repaired. Deep neuromuscular relaxation at this time and no earlier is preferred to minimize airway pressures. Relaxation should be withheld in the presence of concomitant airway anomalies such as tracheomalacia, tracheal pouches, or tracheal agenesis/stenosis, all of which can be identified in advance on bronchoscopy. During operation, both narcotics and volatile anesthetics may be used for maintenance.

## 6. Postoperative Anesthetic Care and Complications

All patients should be admitted to a neonatal intensive care unit after operation. Postoperative fluid maintenance should be guided by parameters such as urine output (target > 1 ml/kg/hr), heart rate, mean blood pressure (targeting the approximate gestational age in weeks), and daily serum sodium, urea, and creatinine.

Early extubation may be an option for nonpremature neonates who have few, if any, associated congenital anomalies and little, if any, respiratory impairment from either aspiration or RDS. Neonates less than 2000 grams will typically require a period of postoperative ventilation [26]. Abrasion to the site of the repaired fistula may be more likely if the trachea remains intubated. On the contrary, laryngoscopy and reintubation if necessary can produce trauma to the fistula site as well as traction on the esophageal anastomosis. Early extubation is not a good option for patients with tracheomalacia or poor lung compliance. It should also not be performed on patients who have underwent thoracotomies, as there are often lung contusions present from the retraction of the ipsilateral lung performed to enable the operative repair. These contusions, which present as opacities on a CXR, can elevate oxygen and pressure requirements for 12–24 hours postoperatively and potentially extend the need for mechanical ventilation.

Enteral nutrition is usually delivered through a feeding tube placed intraoperatively past the esophageal anastomosis, beginning 48 hours postoperatively. Oral feedings may be initiated once the infant can swallow saliva and after a contrast swallow has confirmed the absence of both stenosis and leak at the anastomosis, typically between day 5 and day 7 [21, 22]. Postoperative antibiotics may be given if the newborn is at risk for sepsis due to maternal risk factors such as prolonged rupture of membranes, or if significant esophageal anastomotic leak is likely, based on the integrity of the esophageal tissue and the tension on the anastomosis [16]. For H-type TEF repairs, anastomotic leaks and recurrent fistulas are uncommon, and patients are more likely to tolerate oral feeds. This has been attributed to the absence of an esophageal anastomotic stricture and to a lesser degree of impairment in esophageal motility. Patients may however develop respiratory distress due to tracheal edema, which may be treated with nebulized epinephrine and dexamethasone in older children [30, 31]. Highest incidence of postoperative vocal cord dysfunction is seen after H-type TEF repair [32]. Early vocal cord evaluation should therefore be performed in all H-type TEF patients with respiratory symptoms after their repair. Bilateral cord paralysis may sometimes necessitate tracheostomy due to airway compromise.

The sources of postoperative pain in an infant who has just undergone repair of TEF are the skin incision, the intercostal catheter if a thoracotomy was performed, and the endotracheal tube. Although analgesia for skin incisions should no longer be required in a neonate after 48 hours, analgesia will still be required until the intercostal catheter and endotracheal tube are removed. Regional anesthesia through the caudal catheter can be provided and may facilitate weaning from mechanical ventilation and extubation by reducing the need for opioid consumption. Due to its tendency to decrease systemic vascular resistance, regional anesthesia is contraindicated in infants with coexisting congenital heart disease [25]. Other forms of postoperative analgesia used with success after TEF repairs include intravenous opioid infusions, epidural catheters, subcutaneous wound catheters, and local infiltration. No studies have shown any demonstrable benefit of any one technique over the others [16].

Prior to the first successful repair in 1939, TEF and its associated esophageal atresia (EA) were uniformly fatal. With the advancements in neonatal intensive care, anesthesia, ventilator and nutritional support, and surgical tools and techniques, mortality has become limited largely to neonates suffering from other severe life-threatening anomalies, with survival rates as high as 95% in institutions highly experienced in neonatal health care [21].
